# A continuous fluorescence assay for simple quantification of bile salt hydrolase activity in the gut microbiome

**DOI:** 10.1038/s41598-018-37656-7

**Published:** 2019-02-04

**Authors:** Kristoffer R. Brandvold, Jacqueline M. Weaver, Christopher Whidbey, Aaron T. Wright

**Affiliations:** 1 Chemical Biology & Exposure Sciences, Pacific Northwest National Laboratory, Richland, WA 99352 USA; 2 The Gene and Linda Voiland School of Chemical Engineering and Bioengineering, Washington State University, Pullman, WA 99163 USA

## Abstract

The microbiota of the mammalian gut plays a dynamic role in controlling host physiology. The effect of gut microbiota activity on host health is particularly evident in the case of bile homeostasis. Bile is produced by the host and is modified by the gut microbiota, which impacts the net hydrophobicity of the total bile acid pool, and also modulates host signaling pathways. A key mechanism by which the microbiota modify bile is through deconjugation of bile salts through bile salt hydrolase (BSH) enzymatic activity, which is postulated to be a prerequisite for all further microbial metabolism. BSH activity in the gut is largely considered to be beneficial for the host, and genes encoding BSHs are found in the genomes of many taxa found in over-the-counter probiotics. Despite the therapeutic relevance of this enzyme, there is no sensitive and simple assay for continuous monitoring of BSH activity, and there are no non-destructive means of characterizing its activity in whole cell or microbial community samples. Herein, we describe a continuous fluorescence assay that can be used for characterization of BSH activity with purified protein, cell lysates, whole cells, and in human gut microbiome samples. The method is a “turn-on” reporter strategy, which employs synthetic substrates that yield a fluorescent product upon BSH-dependent turnover. This assay is used to show the first *in vivo* characterization of BSH activity. We also demonstrate continuous, non-destructive quantification of BSH activity in a human fecal microbiome sample containing recombinant BSH.

## Introduction

The animal gut accommodates a complex mixture of microbes that greatly influence host health, with effects including alteration of nutrient absorption, creation of chemical signals that modulate host physiology, and protection against pathogens^1,2^. A major route through which the gut microbiome affects the host is through chemical transformations of xeno- and endobiotics^3^. The capacity for certain types of chemical transformations depends on the identity and abundance of the individual microbes within the gut microbiome, which can vary dramatically between individuals, and are influenced by factors including diet, age, geographic location, and even season^4^. The metabolic activity of the gut microbiome can positively affect host health through increasing the nutritional value that can be extracted from food, as in the case of digestion of complex polysaccharides^5^. The gut microbiome can also create chemical metabolites, such as short chain fatty acids, which induce signal transduction pathways in the host that reduce susceptibility to disease^6^. Alternatively, chemical modification of host-produced metabolites by the gut microbiota can be detrimental, as some metabolites are associated with increased incidence of cancer^7^. A clearer understanding of gut microbiome metabolism is needed to increase host resilience and decrease susceptibility to disease.

A well-established example of a microbiota-host interaction is the microbial modification of the host’s bile fluid^8,9^. Animals produce bile to aid in the digestion of hydrophobic nutrients through increasing solubility. Bile acids, which are small organic compounds that are a primary component of bile, have a large effect on the net physicochemical properties of this fluid. Bile acids are produced in the liver through a multi-step biosynthetic pathway, which uses cholesterol as a starting material^10^. Humans produce two primary bile acids, cholic and chenodeoxycholic acid^11^, which differ by the presence of one hydroxyl group, but can have distinct biological effects. After synthesis in the liver, bile acids are subsequently conjugated to glycine or taurine to yield bile salts, which significantly increases the hydrophilicity of the molecule. After synthesis in the liver, bile salts are stored in the gall bladder, and are released into the duodenum to facilitate digestion, and are eventually reabsorbed for later use^12^.

Although bile acids clearly influence digestion, these molecules are no longer thought of as simple surfactants that increase solubility of hydrophobic nutrients, but are now also appreciated to be selective chemical signals that bind human nuclear receptors in the intestine such as FXR^13,14^ and the vitamin D receptor^15^, and cell surface receptors such as TGR5^16,17^. Binding of bile salts by host receptors provides information on the state of the bile acid pool, and negatively regulates the first step in the synthesis of bile salts from cholesterol. Bile acid receptors also feed into signaling pathways that regulate other processes, including glucose and energy homeostasis^18^.

**Figure 1:**
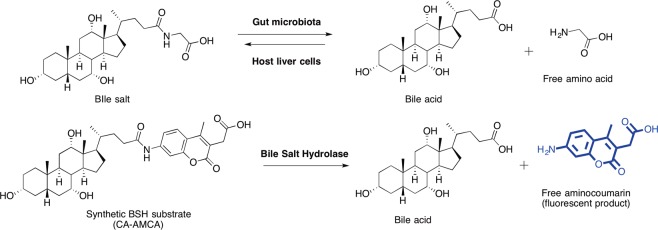
Design of an assay to quantify the activity of an enzyme that is a key regulator of bile acid homeostasis. (**A**) Bile salt hydrolase activity of the gut microbiota modulates the properties of bile salts, which are produced by the liver. BSH facilitates hydrolysis of the amide bond between the bile acid and glycine/taurine. (**B**) Chemical structures for cholic acid–AMCA probe (CA-AMCA), and proposed strategy for continuous monitoring of BSH activity. The probe was created by a one-step chemical synthesis in which a purified bile acid was coupled to 7-amino-4-methyl-3-coumarinylacetic acid, and subsequently purified by reverse-phase HPLC.

Microbial metabolism in the gut produces modified, secondary bile acids including deoxycholic, ursodeoxycholic, and lithocholic acid^19^, which can have disparate biological effects relative to each other and the primary bile acids^20^. Bile salts must be deconjugated from glycine/taurine in order for the microbiota to modify primary bile acids^21^. Deconjugation of bile salts results from the activity of microbial bile salt hydrolases (BSHs), which hydrolyze the amide bond between the bile acid and glycine/taurine (Fig. 1A) using a nucleophilic cysteine^3,22^. Because of its role in preparation of bile acids for further microbial modification, BSH can be thought of as the gatekeeper for secondary bile acid formation. The result of BSH deconjugation activity is a net change in the physicochemical properties of the bile acid pool, which ultimately makes it more hydrophobic. BSH deconjugation is also strongly correlated with excretion of bile acids^23^. Increasing BSH activity has been suggested to be an effective strategy for treating obesity and hypercholesterolemia, because if bile acids are excreted in excess, endogenously produced cholesterol pools will be depleted to compensate for the production of fresh bile^13^. BSH activity has been proposed to be responsible for the beneficial health effects of several common probiotics^24^.

Enzymatic hydrolysis of bile salts by BSH generally relies on a N-terminal nucleophilic cysteine that attacks the amide bond of a bile salt to free the amino acid and form an intermediate covalent enzyme-bile acid complex, which is subsequently hydrolyzed, and leaves the cysteine free to participate in another catalytic cycle^3,22^. Current methods for quantification of BSH activity rely on multi-step detection of free amines^25,26^ (amino acid product), HPLC purification^27^, or through spectrophotometric detection of precipitated products^28,29^. These methods all suffer from respective drawbacks. Detection of free amines, as in ninhydrin-based assays, is not practical when analyzing complex samples (e.g. cell lysates or stool) that contain many nucleophilic amines that would cause assay interference. HPLC methods suffer from lengthy experiment times and low-throughput data generation. Crucially, few of the current methods are useful for analyzing BSH activity in fecal samples, which are most relevant to a clinical setting. Additionally, there are currently no methods that can be applied in *in vivo* bacterial cultures, which significantly limits the types of questions that researchers can ask about the biology of BSH. Herein, we describe a reliable, simple assay that addresses all of these issues.

Inspired by the success of aminocoumarin-peptide conjugate reporters of protease activity^30–32^, it was envisioned that a similar approach could be applied to BSH, which also acts as an amide hydrolase. We implemented this approach to design a chemical probe that is a conjugate of cholic acid and an aminocoumarin fluorophore (CA-AMCA; Fig. 1B). Cholic acid was chosen for this study because its salts are reported to have slightly better BSH turnover rates than the analogous chenodeoxycholic acid conjugates^3^. Based upon the known properties of peptide-aminocoumarin conjugates, we rationalized that aminocoumarin fluorescence could be decreased through amide coupling of the amine to a carboxylic acid of a bile acid, and that upon exposure of the synthetic substrate to a BSH, the amide bond would be cleaved and produce a free bile acid and the unquenched free aminocoumarin fluorophore. Product formation could therefore be continuously and quantitatively monitored using a fluorimeter. Fluorescent bile acid analogs have been previously reported^33–36^, and were used for characterization of bile acid movement in the gut or other applications, but have not been applied to a kinetic activity-based assay for BSH.

## Results and Discussion

### Chemical synthesis and spectroscopic characterization of CA-AMCA

**Figure 2:**
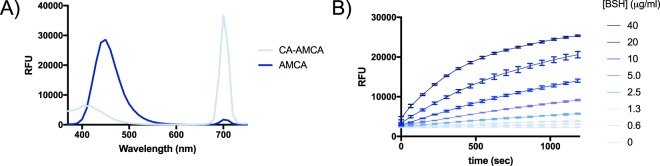
AMCA fluorescence is greatly decreased upon coupling to cholic acid, and the product (CA-AMCA) continuously reports on activity of purified recombinant BSH in buffer. (**A**) Solutions of 150 μM AMCA and 150 μM CA-AMCA in PBS were characterized to determine emission properties when excited at 350 nm. (**B**) CA-AMCA (150 μM) was added to buffered solutions with varying concentrations of BSH. Increasing the concentration of BSH resulted in increased rates of CA-AMCA hydrolysis as assessed by monitoring product formation using a fluorescence plate reader. Standard deviation for n = 3 replicates is shown superimposed on line that connects the measured averages.

We synthesized our probe through amide coupling of cholic acid with 7-amino-4-methyl-3-coumarinylacetic acid (AMCA) using the standard HATU coupling reagent in dimethylformamide with Hünig’s base (See SI for a complete description). The product was isolated via reverse-phase high pressure liquid chromatography. Spectrographic characterization of the product revealed that, similar to the analogous protease substrates, the conjugated probe was much less fluorescent relative to the free aminocoumarin when examined at identical excitation and emission wavelengths (Fig. 2A), and thus had potential to act as a reporter of BSH activity.

### Biochemical characterization of CA-AMCA with purified recombinant BSH

When the CA-AMCA probe was added to buffer containing purified BSH, a fluorescence plot consistent with enzymatic turnover of the synthetic substrate was observed (Fig. 2B, SI Fig. 1). No increase in fluorescence was observed when the probe was added to buffer alone or to buffer containing an unrelated hydrolase (SI Fig. 2), implying that the probe is stable in aqueous solutions and has selectivity for BSH. Fluorescent signals for substrate turnover could be detected with as low as mid-nanomolar enzyme concentrations, which is considerably less enzyme when compared to micromolar concentrations required for conventional ninhydrin assays with purified BSH^3^. Product formation was additionally confirmed through coupling of the BSH reaction to a secondary HPLC-based assay (SI Fig. 3).

**Figure 3:**
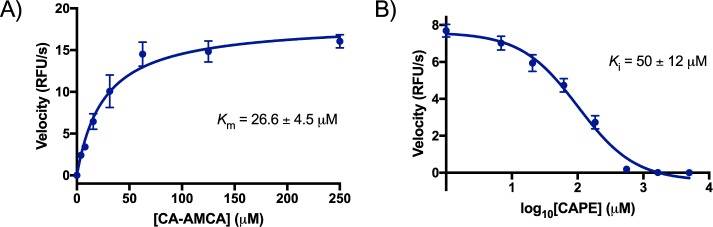
Biochemical characterization of a synthetic BSH substrate (CA-AMCA). (**A**) Substrate affinity was assessed through generation of a Michaelis-Menten curve by treating a buffered solution of purified BSH with varying concentrations of CA-AMCA and plotting the turnover rates against substrate concentration, and (**B**) *K*_i_ for caffeic acid phenylethyl ester (CAPE), which is a previously reported small molecule inhibitor of BSH.

Attenuated activity was observed when a large amount of the native substrate, glycocholic acid, was added to the reaction (SI Fig. 4), which indicates that the probes bind the canonical BSH substrate-binding site. Addition of iodoacetamide, which covalently caps nucleophilic cysteines, completely ablated substrate turnover, which suggests that the probes are being hydrolyzed in a cysteine-dependent mechanism that is consistent with native substrate hydrolysis (SI Fig. 4). After confirming that CA-AMCA was behaving similarly to a native substrate, we then sought out to quantitatively analyze the affinity of CA-AMCA for BSH, and further biochemical characterization revealed a low two-digit micromolar Michaelis-Menten constant (*K*_m_ = 26 μM; Fig. 3A and SI Fig. 5), which is lower than reported values for native BSH substrates^37^.

Selective pharmacological modulators of BSH would have applications in human medicine^24^ and veterinary applications^38^. At least one previous study has uncovered small molecule BSH modulators through screening of a small molecule library^39^, but assay reliance on quantification of bile acid precipitation relegated this study to a relatively small compound library (<2,500). Furthermore, previous activity-based assays for BSH are incompatible with either mono-cultures of living cells or multi taxa-containing gut microbiome samples. It was postulated that the CA-AMCA reporter would be especially well-suited for characterization of small molecule BSH modulators in protein, whole cell, and microbial community samples. The CA-AMCA probe provided a *K*_i_ value for the BSH inhibitor, caffeic acid phenylethyl ester (CAPE)^39^, that is in good agreement with previous reports that used conventional BSH assays (Fig. 3B, SI Fig. 6).

### Characterization of CA-AMCA in cell lysates and whole cells

After establishing that the CA-AMCA reporter functions reliably with purified protein, the possibility of analyzing complex biological mixtures was explored through addition of known amounts of BSH to a lysate of *E. coli* cells, which do not express BSH (SI Fig. 7). Rates of substrate turnover correlated with BSH concentration and were comparable to the results obtained with purified protein, suggesting that this assay accurately reports on BSH activity in complex biological samples.

To our knowledge, there is currently no quantitative method to continuously and non-destructively monitor bile salt hydrolase activity *in vivo*, and so we sought to evaluate our CA-AMCA reporter to this end. *Lactobacillus plantarum* is a well-characterized gut symbiont that expresses four forms of BSH^40^. This species is of particular interest because it is a commonly ingested probiotic, which has shown to positively impact hypercholesterolemia. Therefore, *L. plantarum* was employed as a model organism for *in vivo* characterization of the CA-AMCA probe.

**Figure 4:**
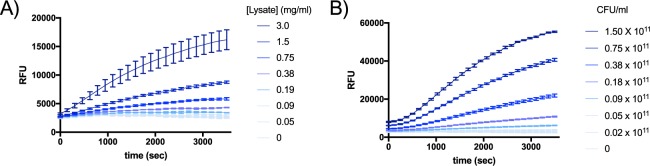
The CA-AMCA assay is capable of detecting endogenously expressed BSH both in cell lysates and *in vivo* for *L. plantarum*, which is a BSH-expressing habitant of human gut. Standard deviation for n = 3 replicates are shown superimposed on line that connects the measured averages. (**A**) CA-AMCA (150 μM) was added to buffered solutions containing varying amounts of *L. plantarum* lysate. (**B**) CA-AMCA (150 μM) was added to buffered solutions containing varying amounts of intact *L. plantarum* cells. Rates of CA-AMCA turnover are dependent on cell density.

First, *L. plantarum* cell lysate was treated with CA-AMCA, and substrate turnover was observed as expected (Fig. 4A, SI Fig. 8). Activity was not observed when lysate was treated with CAPE, suggesting that the observed activity is due to BSH (SI Fig. 9). No substrate turnover was observed when the probe was added to a cell lysate of a microbe (*E. coli)* that does not express BSH (SI Fig. 7). Second, CA-AMCA was evaluated in whole-cell monocultures of *L. plantarum*. Cells were grown until exponential phase and suspended in buffer for analysis. Addition of CA-AMCA to a suspension of live *L. plantarum* cells yielded fluorescence plots that are consistent with substrate turnover (Fig. 4B, SI Fig. 10). No activity was observed when the probe was added to a suspension of living microbes that do not express BSH (SI Fig. 11). We believe this to be the first non-destructive whole-cell analysis of BSH activity.

### Characterization of CA-AMCA in human fecal microbiome samples

**Figure 5:**
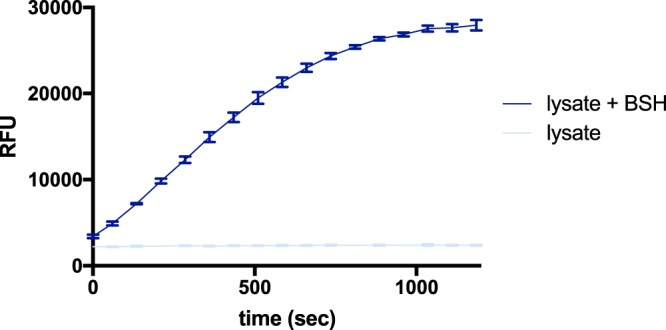
CA-AMCA robustly reports on BSH activity in the context of human fecal microbiome lysates. CA-AMCA (150 µM) was added to a prepared sample of buffered human gut microbiome lysate (500 µg/ml protein) with purified BSH (20 µg/ml) added, and substrate turnover was monitored using a fluorescence plate reader. Standard deviation for n = 3 replicates are shown superimposed on line that connects the measured averages.

This assay would be especially beneficial for the characterization of BSH activity in gut microbiome extracts from human fecal samples because there are currently no simple methods for this. Such a method could be valuable for clinicians to quantify net BSH activity in patient samples. So, it was next determined if the CA-AMCA reporters could function in complex gut microbiome samples. For our representative gut microbiome model, we used a human fecal sample that was purchased from a commercial vendor (LeeBio Solutions). The cell fraction was isolated via centrifugation and subsequently subjected to lysis. Purified BSH was added to a human fecal microbiome sample from a single donor, and a proportionate increase in substrate turnover was observed (Fig. 5, SI Fig. 12). This is the first demonstration of continuous quantitative analysis of BSH activity in a human fecal microbiome sample. Promisingly, we also observed signal from endogenously expressed BSH in *L. plantarum* lysate when it was combined with fecal microbiome lysate (SI Fig. 13).

In conclusion, the metabolic chemistry of the gut microbiome is becoming increasingly appreciated to be a major determinant of host health, and new approaches are required to define the scope and magnitude of these processes. We have developed a new strategy to continuously monitor the microbial enzyme bile salt hydrolase, which primes bile acids for further microbial metabolism, and is proposed to be the major player in the positive health effects of many over-the-counter probiotics. This assay is the first example of real-time continuous monitoring of BSH activity, and the approach has low experimental variability (SI Figs 14 and 15). It is also demonstrated for the first time that BSH activity can be monitored using this approach in whole cells and also in the presence of human fecal microbiome homogenate. We envision that this approach will be beneficial for clinicians that are seeking to quantify BSH activity in patient-derived samples. We also see this as a superior strategy to discovery small molecule modulators of BSH because this system is applicable to both whole cell and gut microbiome samples. As the tools to study the microbiome become increasingly refined, so will our ability to diagnose and hopefully treat the myriad diseases that are influenced by the microbiota-host relationship.

## Materials and Methods

### Chemical synthesis of CA-AMCA

Details for chemical synthesis and characterization of CA-AMCA can be found in the Supplementary Information.

### Cell lines and media

*Lactobacillus plantarum* (Orla-Jensen) was purchased from ATCC (ATCC BAA-793) and cultured according to the vendor’s recommendations using MRS media (Fisher Scientific, Difco Lactobacilli MRS Broth, BD288130). ElectroMAX DH5α-E Competent Cells were purchased from ThermoFisher (11319019) and were cultured in LB media (FisherScientific, AC612725000) according to the vendor’s recommendations. See SI for complete description of cell lines and culture conditions.

### Representative assay conditions for purified protein

(See SI for description of all assays) Reaction volumes of 100 µL were used in Microfluor1 black flat bottom microtiter 96 well plates (Thermo Scientific). 50 μl BSH (80, 40, 20, 10, 5, 2.5, 1.3, 0 µg/ml) in 0.1 M sodium phosphate buffer (pH = 6) were added to the appropriate wells. A 50 µL solution of probe (300 µM) in buffer with 5% DMSO was then added to initiate the reaction. Reactions were immediately placed in a plate reader (SPECTRAmax Gemini XS) pre-warmed to 37 °C, and reaction progress was monitored at 450 nm (excitation 350 nm) for 20 minutes. Reactions had final concentrations of 150 μM probe and 2.5% DMSO.

### Representative assay conditions for cell lysates

(See SI for description of all assays.) Reaction volumes of 100 µL were used in Microfluor1 black flat bottom microtiter 96 well plates (Thermo Scientific). 50 µL of *L. plantarum* lysate (3000, 1500, 750, 375, 188, 94, 47 μg/ml) in buffer (0.1 M sodium phosphate, pH = 6) was added to each well. A 50 µL solution of probe (300 µM) in buffer with 5% DMSO was then added to initiate the reaction. Reactions were immediately placed in a plate reader (SPECTRAmax Gemini XS) pre-warmed to 37 °C, and reaction progress was monitored at 450 nm (excitation 350 nm) for 20 minutes. Reactions had final concentrations of 150 μM probe, 0.1 M sodium phosphate (pH 6), and 2.5% DMSO.

### Representative assay conditions for whole cells

(See SI for description of all assays.) A starter culture was created by inoculation of 5 ml MRS broth from a L. plantarum colony grown on MRS agar. The starter culture was incubated overnight at 37 °C overnight. The 5 ml starter culture was then added to 95 ml fresh MRS broth and grown until the optical density at 600 nm reached 0.7. At that point, the cells were pelleted via centrifugation. The cell pellet was resuspended in 15 ml PBS, and centrifuged. The cells were additionally washed with PBS and pelleted once more. The resulting pellet was suspended in 380 μl PBS. The cell suspension was then serially diluted two-fold.

50 μl of the cell suspension was added to Microfluor1 black flat bottom microtiter 96 well plates (Thermo Scientific). A 50 µL solution of probe (300 µM) in buffer with 5% DMSO was then added to initiate the reaction. Reactions were immediately placed in a plate reader (SPECTRAmax Gemini XS) pre-warmed to 37 °C, and reaction progress was monitored at 450 nm (excitation 350 nm) for 60 minutes. Reactions had final concentrations of 150 μM probe and 2.5% DMSO.

### Preparation of human fecal microbiome cell lysate

Human fecal sample from a single donor was obtained from Lee BioSolutions (Cat. No. 991-18). Sample (1 g) was suspended in 15 mL PBS with glass beads (3 mM) by vortexing for 30 seconds. The suspension was then allowed to rest on ice for 10 minutes to settle beads and large debris. The supernatant was transferred to a clean 50 mL conical tube. The supernatant was centrifuged at 700 *g* for 15 minutes at 4 °C. The supernatant was transferred to a clean 15 mL conical tube. The supernatant was then centrifuged at 7000 *g* for 15 minutes at 4 °C to pellet the bacterial cells. The supernatant was then discarded. The cell pellet was resuspended in 1 mL cold PBS, and transferred to a 1.7 mL Eppendorf tube. The sample was vortexed briefly to homogenize the solution. The cells were then pelleted at 8000 *g*. The pellet was then washed in 1 mL cold PBS. The pellet was then resuspended in 2 mL of PBS. Samples were transferred to 1.5 mL SafeLock tubes. 50–100 µL of small (0.3 mm) glass beads were added. Samples were lysed in a Bullet Blender bead mill homogenizer (Next Advance; Model: BBX24B-CE) at max speed for 5 minutes. Bead beating was repeated two more times for a total of 3 rounds. Protein content was then determined by BCA assay (Thermo Scientific).

### Analysis of BSH activity in a gut microbiome sample

Reaction volumes of 100 µL were used in Microfluor1 black flat bottom microtiter 96 well plates (Thermo Scientific). 50 μl of gut microbiome lysate (1 mg/ml) with BSH (40 µg/ml) or buffer were added to the appropriate wells. A 50 µL solution of probe (300 µM) in buffer with 5% DMSO was then added to initiate the reaction. Reactions were immediately placed in a plate reader (SPECTRAmax Gemini XS) pre-warmed to 37 °C, and reaction progress was monitored at 450 nm (excitation 350 nm) for 20 minutes. Reactions had final concentrations of 20 µg/ml BSH, 500 µg/ml lysate protein, 150 μM probe and 2.5% DMSO.

## Supplementary information

SI

## Data Availability

Individual replicate data from fluorescence studies will be provided upon request.
