# Supplementary material for: A continuous fluorescence assay for simple quantification of bile salt hydrolase activity in the gut microbiome
Source: Sci Rep. 2019 Feb 4;9:1359. doi: 10.1038/s41598-018-37656-7 (PMC6361941; doi:10.1038/s41598-018-37656-7)
Supplement: Supplementary file 1 — SI [file 41598_2018_37656_MOESM1_ESM.pdf]

SUPPLEMENTARY INFORMATION

A continuous fluorescence assay for simple quantification of bile salt hydrolase activity in the gut microbiome.

Kristoffer R. Brandvold[a], Jacqueline M. Weaver[a], Christopher Whidbey[a], and Aaron T. Wright[a,b]*

[a] Chemical Biology & Exposure Sciences, Pacific Northwest National Laboratory, Richland, WA 99352 (USA)

[b] The Gene and Linda Voiland School of Chemical Engineering and Bioengineering, Washington State University, Pullman, WA 99163 (USA)

E-mail: aaron.wright@pnnl.gov

TABLE OF CONTENTS

I.	Supplementary Data for Manuscript	S3-S18
II.	Supplemental Experimental Procedures	S19-S21
i.	General synthetic methods	S19
ii.	Synthetic procedures	S19
iii.	Spectral data	S20
iv.	Expression, purification and characterization of recombinant BSH	S21
v.	Biological characterization of probes	S21-S24

I. SUPPLEMENTARY DATA FOR MANUSCRIPT

A)

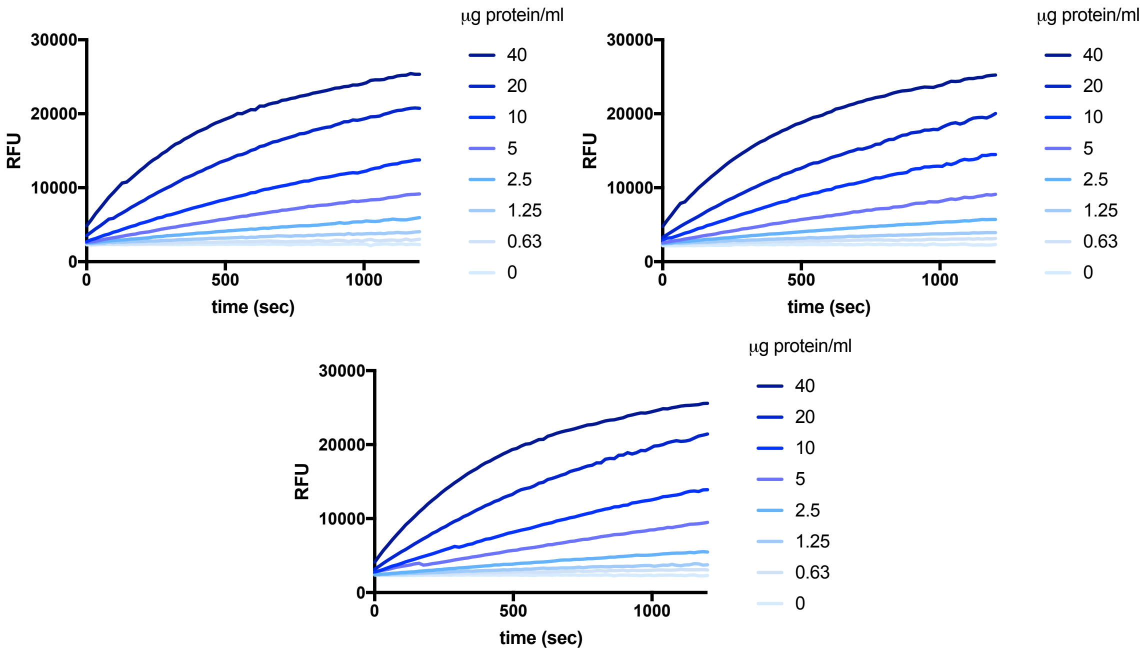

B)

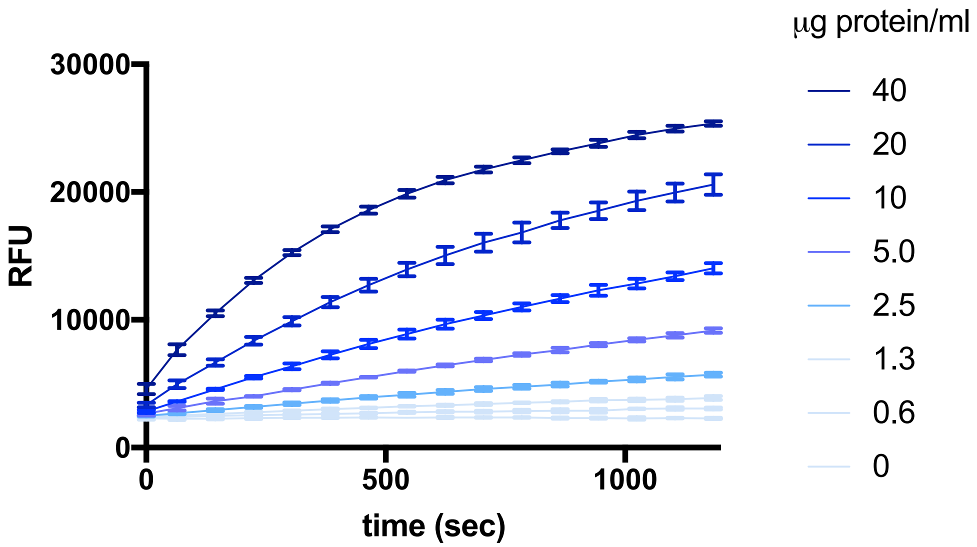

SI Figure 1. Fluorescence increases proportionately with increasing BSH concentration in CA-AMCA assay. A) Individual replicates for titration of purified BSH. B) Averages for selected timepoints with standard deviations shown (n = 3 replicates).

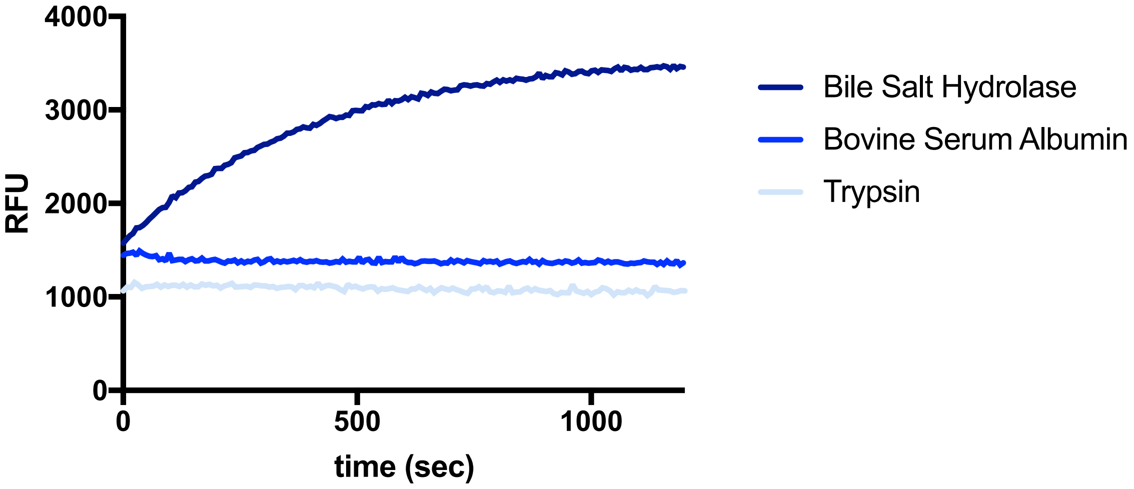

SI Figure 2. CA-AMCA reacts with BSH, but not BSA or an unrelated hydrolase. Treatment of purified protein with CA-AMCA. Solutions of given protein were treated with 150 μM CA-AMCA and reaction kinetics were determined through monitoring of fluorescence at 450 nm when excited at 350 nm.

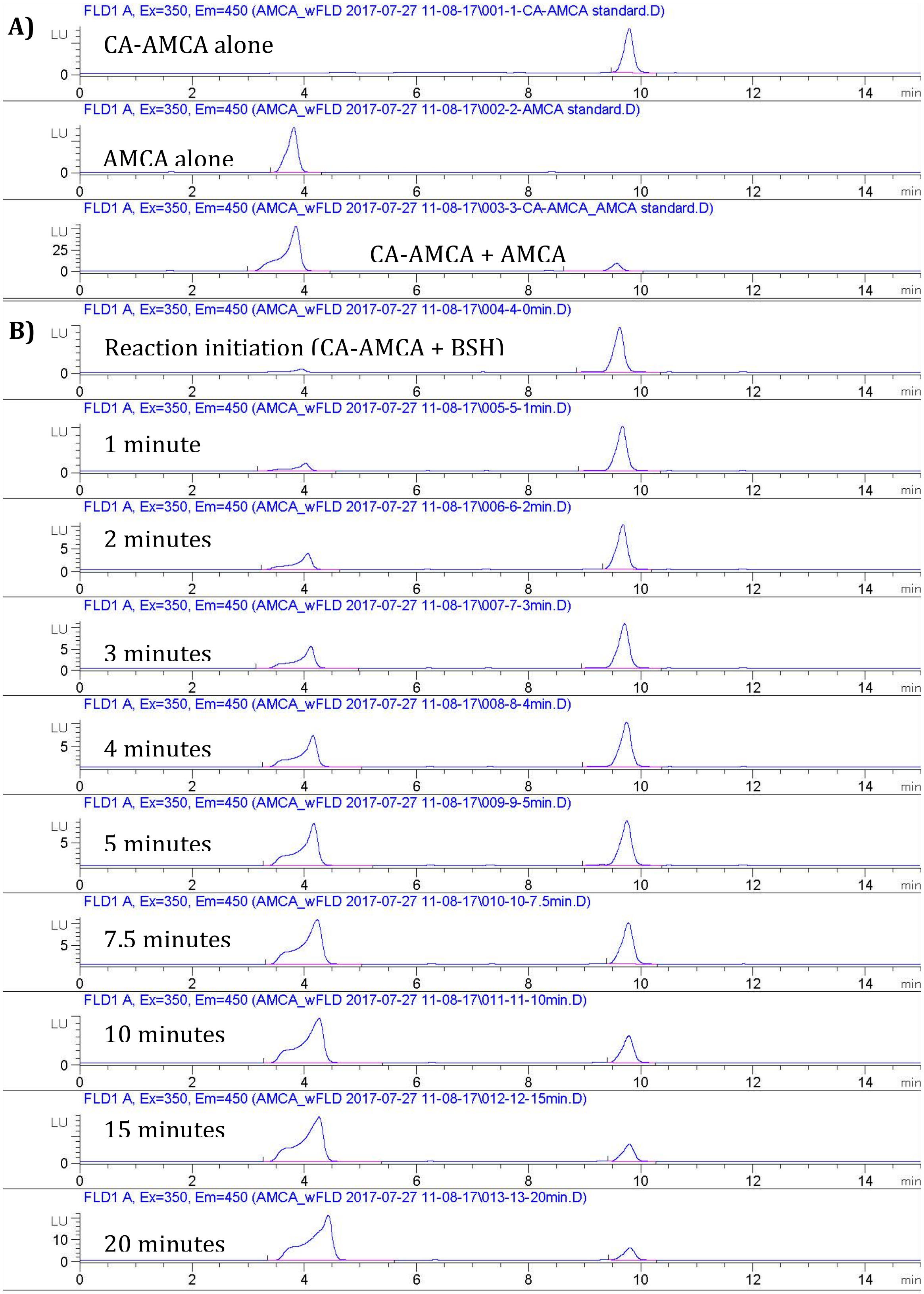

SI Figure 3. HPLC analysis of reaction of CA-AMCA with BSH provides results that are consistent with hydrolytic production of AMCA. Purified CA-AMCA and AMCA

were first subjected to RP-HPLC and fluorescence was monitored (Ex:350, Em:450. B) BSH was treated with 150 μM CA-AMCA, and at the specified time points aliquots were removed from the reaction and quenched with iodoacetamide. The quenched reactions were then subjected to RP-HPLC.

A)

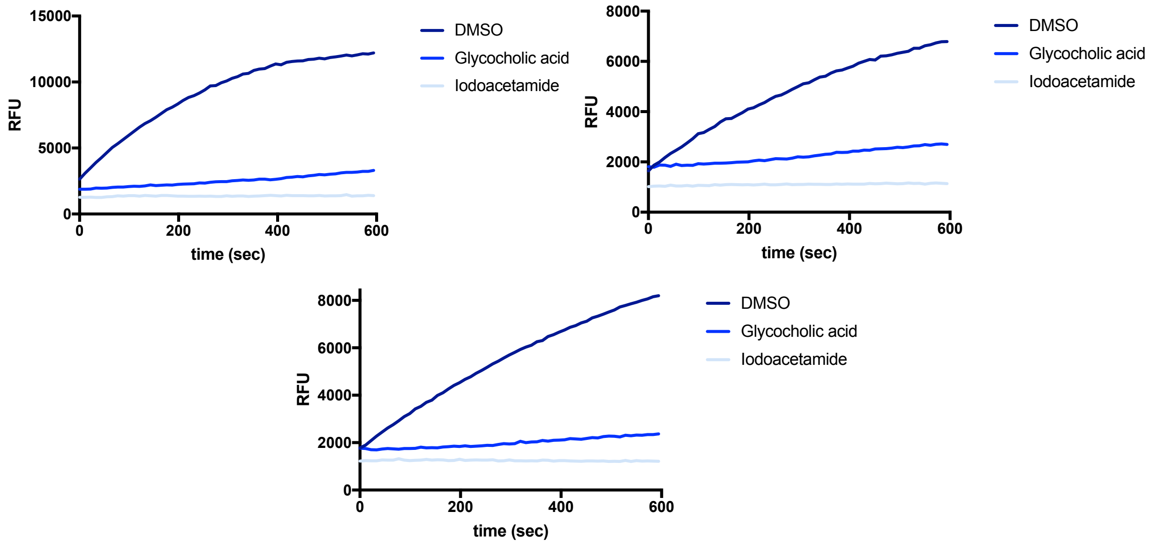

B)

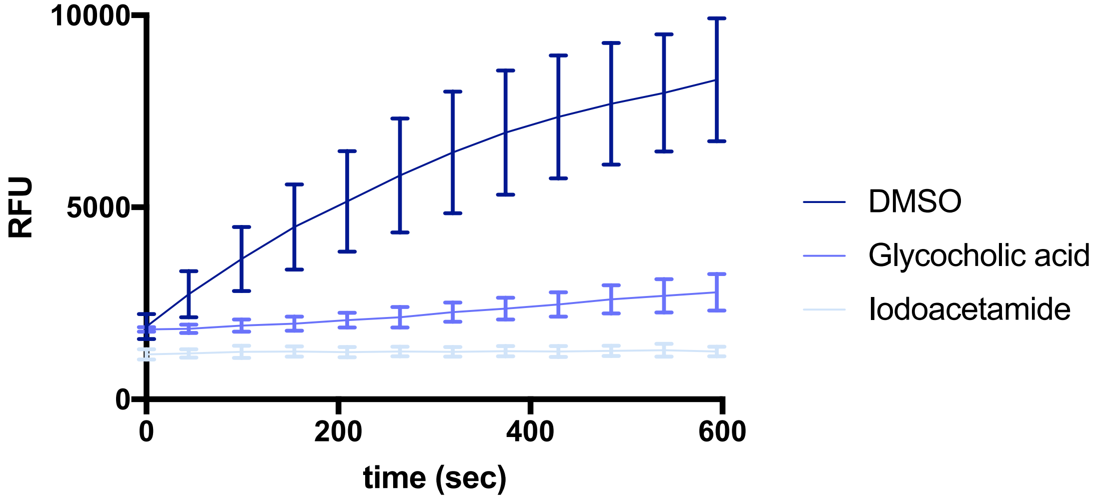

SI Figure 4. CA-AMCA probe binds to canonical BSH substrate-binding site, and is turned over in a cysteine-dependent manner. A) Individual replicates for purified BSH treated with iodoacetamide or glycocholic acid prior to CA-AMCA addition. B) Averages for selected timepoints with standard deviations shown (n = 3 replicates).

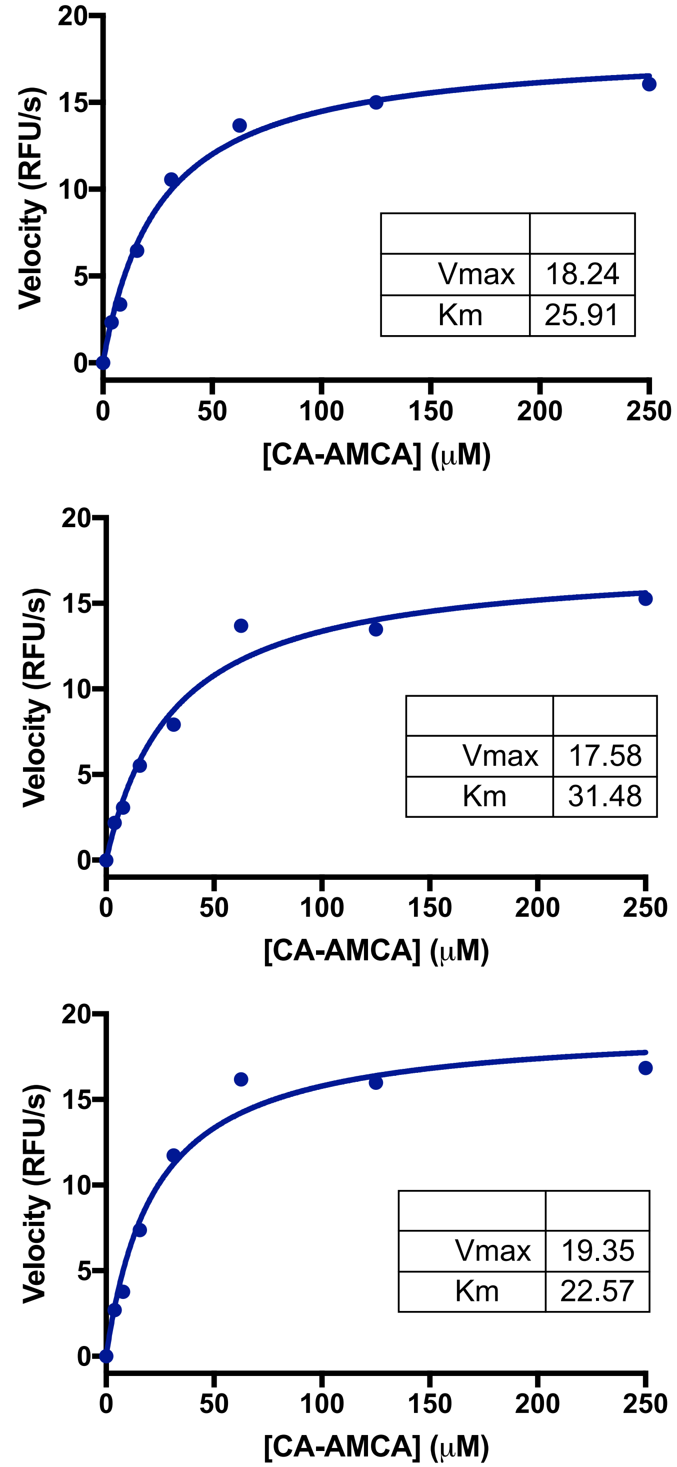

SI Figure 5. Individual Km curves for CA-AMCA probe show affinity is similar to reported values for native substrates. BSH was treated with 150 μM CA-AMCA, and substrate turnover was assessed through monitoring of fluorescence at 450 nm when excited at 350 nm. Initial velocities were recorded at several probe concentrations and plotted as a K_m curve.

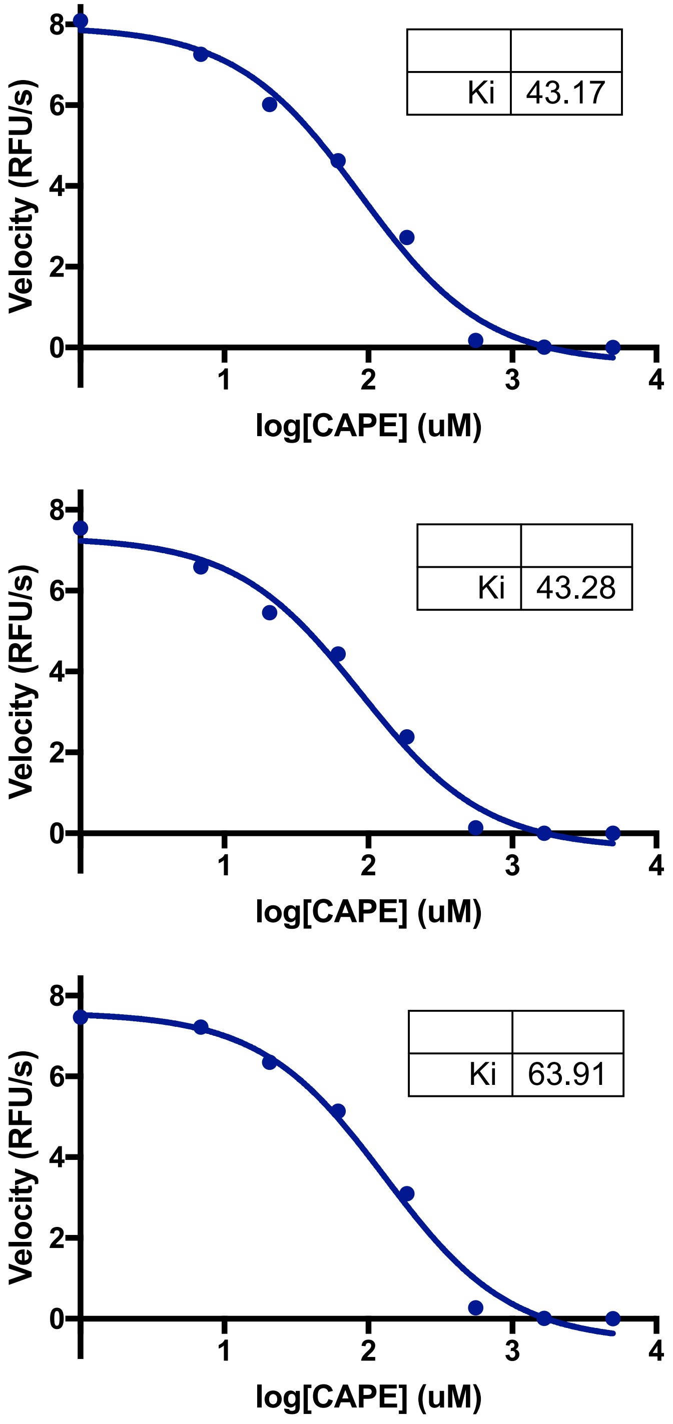

SI Figure 6. CA-AMCA assay can be used for characterization of small molecule modulators of BSH. Individual replicates for CAPE Ki determination.

A)

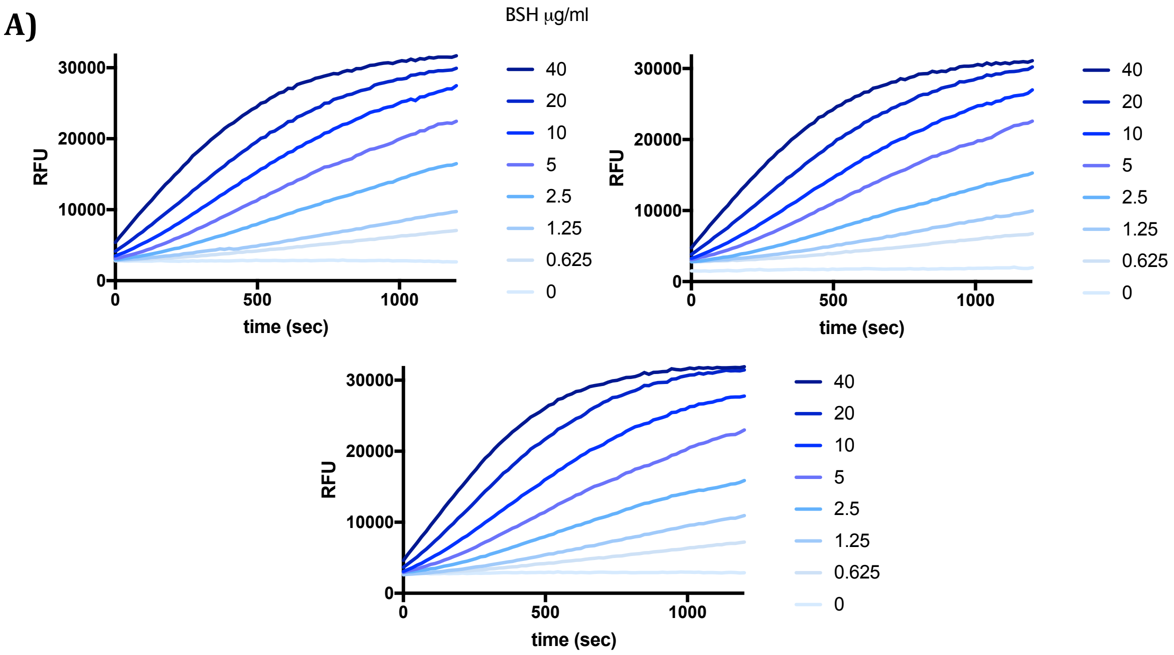

B)

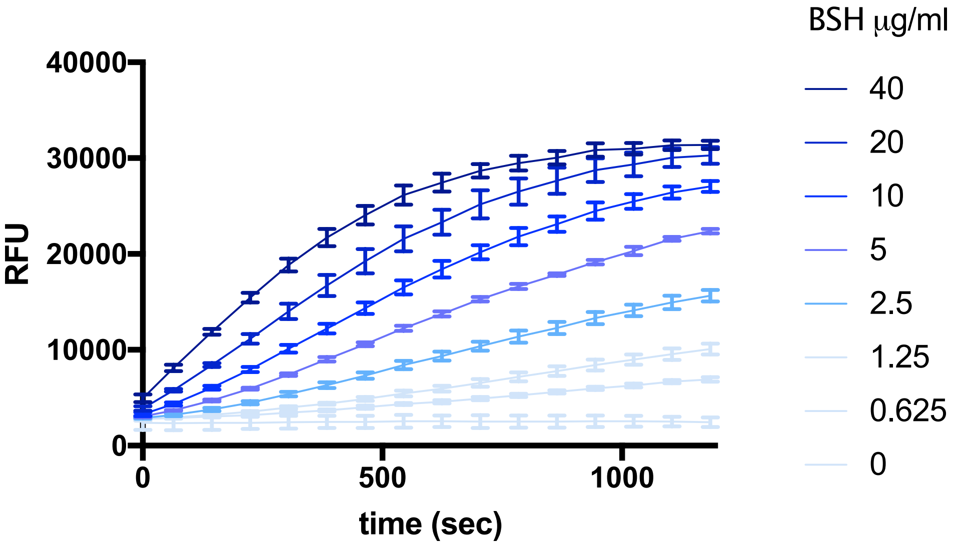

SI Figure 7. CA-AMCA assay is capable of reporting on BSH activity in cell lysates. A) Individual replicates for addition of purified BSH to *E. coli* lysate. B) Averages for selected timepoints with standard deviations shown (n = 3 replicates).

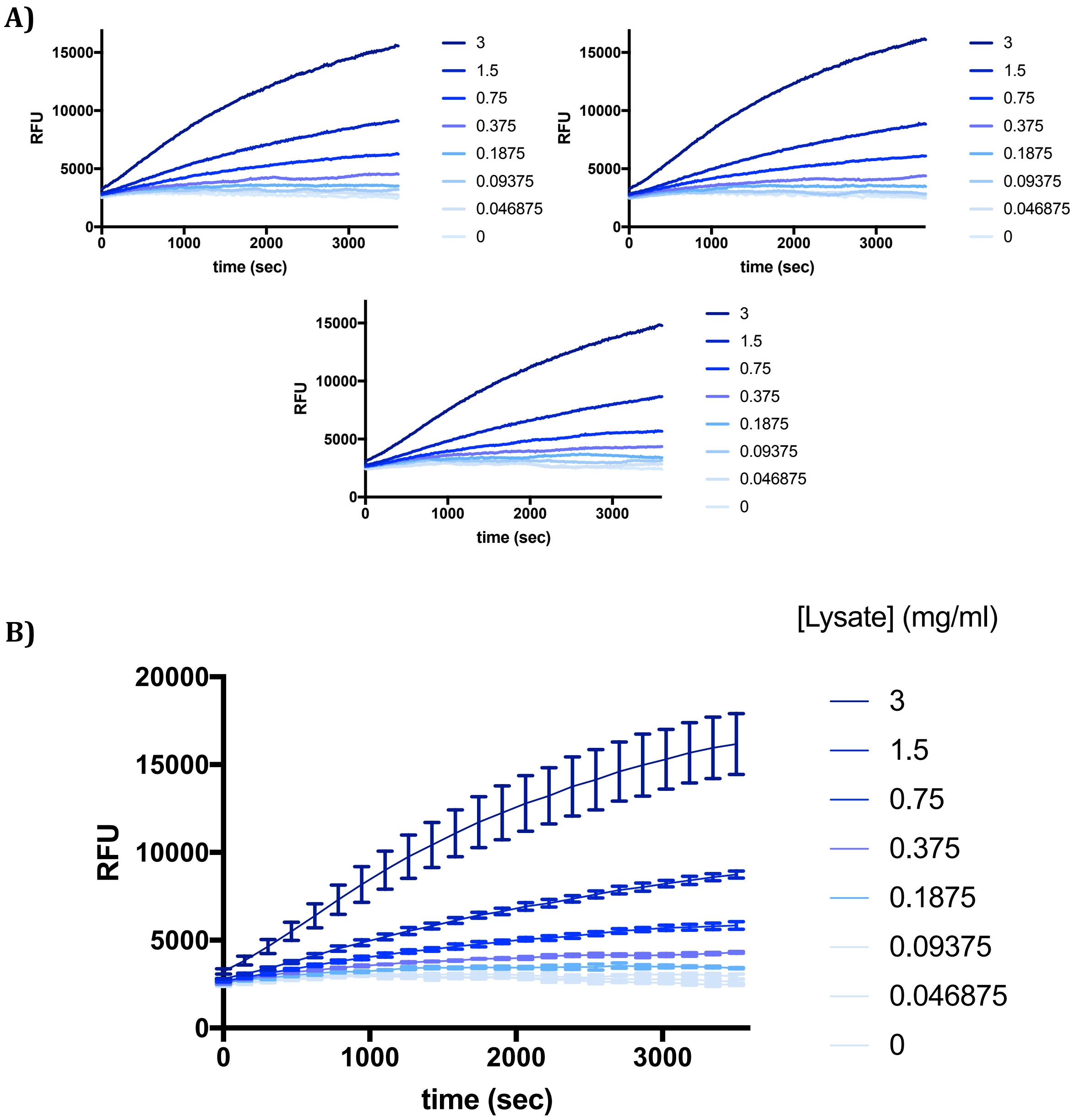

SI Figure 8. CA-AMCA reports on activity of endogenously expressed BSH in L. plantarum lysate. Varying concentrations of *L. plantarum* lysate were treated with 150 μM CA-AMCA and reaction kinetics were monitored through fluorescence quantification. A) Individual replicates. B) Averages for selected timepoints with standard deviations shown (n = 3 replicates).

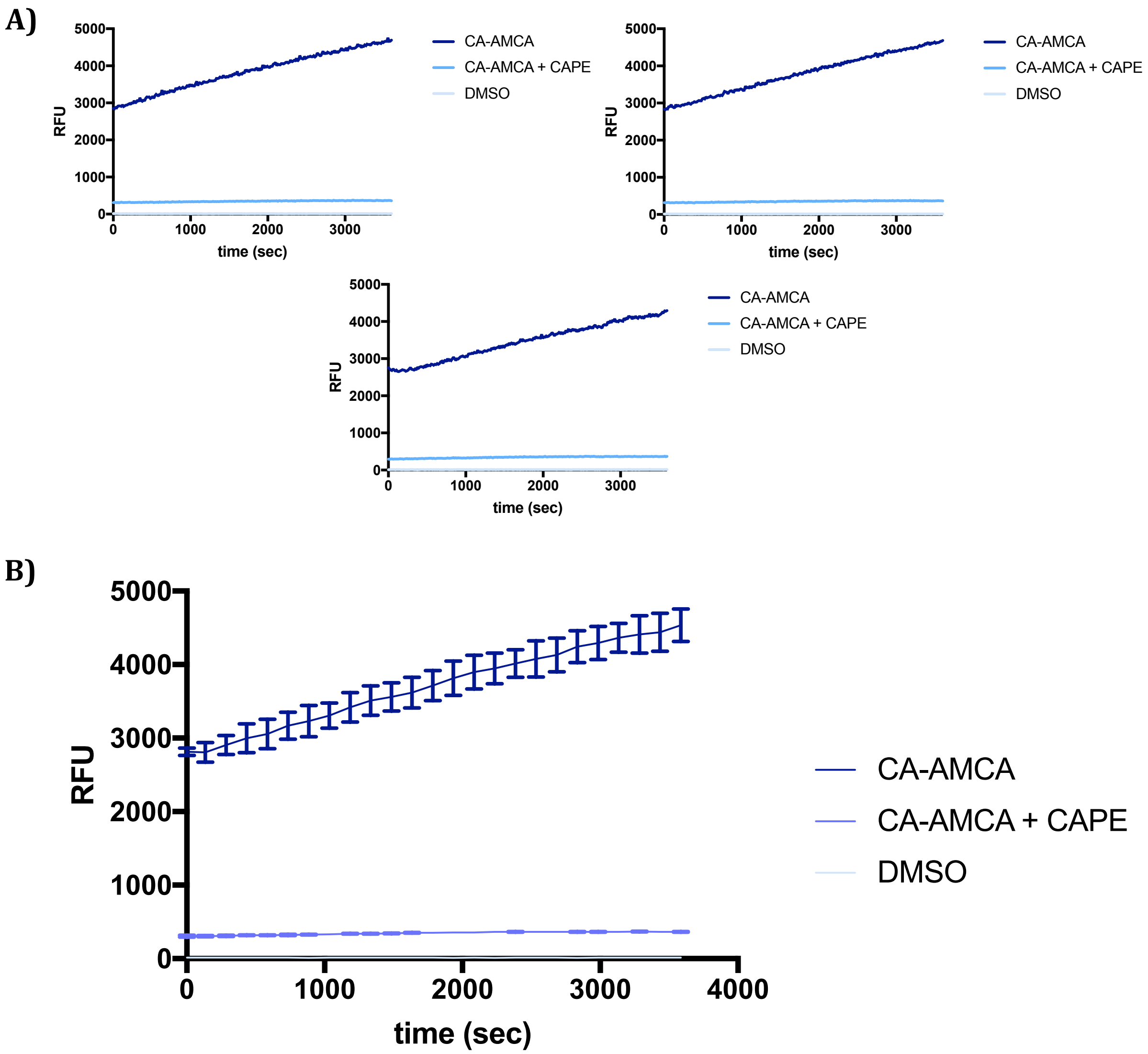

SI Figure 9. Pharmacological inhibition confirms that observed activity is due to BSH. No substrate turnover is observed in *L. plantarum* lysate when the BSH inhibitor, CAPE (2 mM), is added to the reaction. A) Individual replicates. B) Averages for selected timepoints with standard deviations shown (n = 3 replicates).

A)

B)

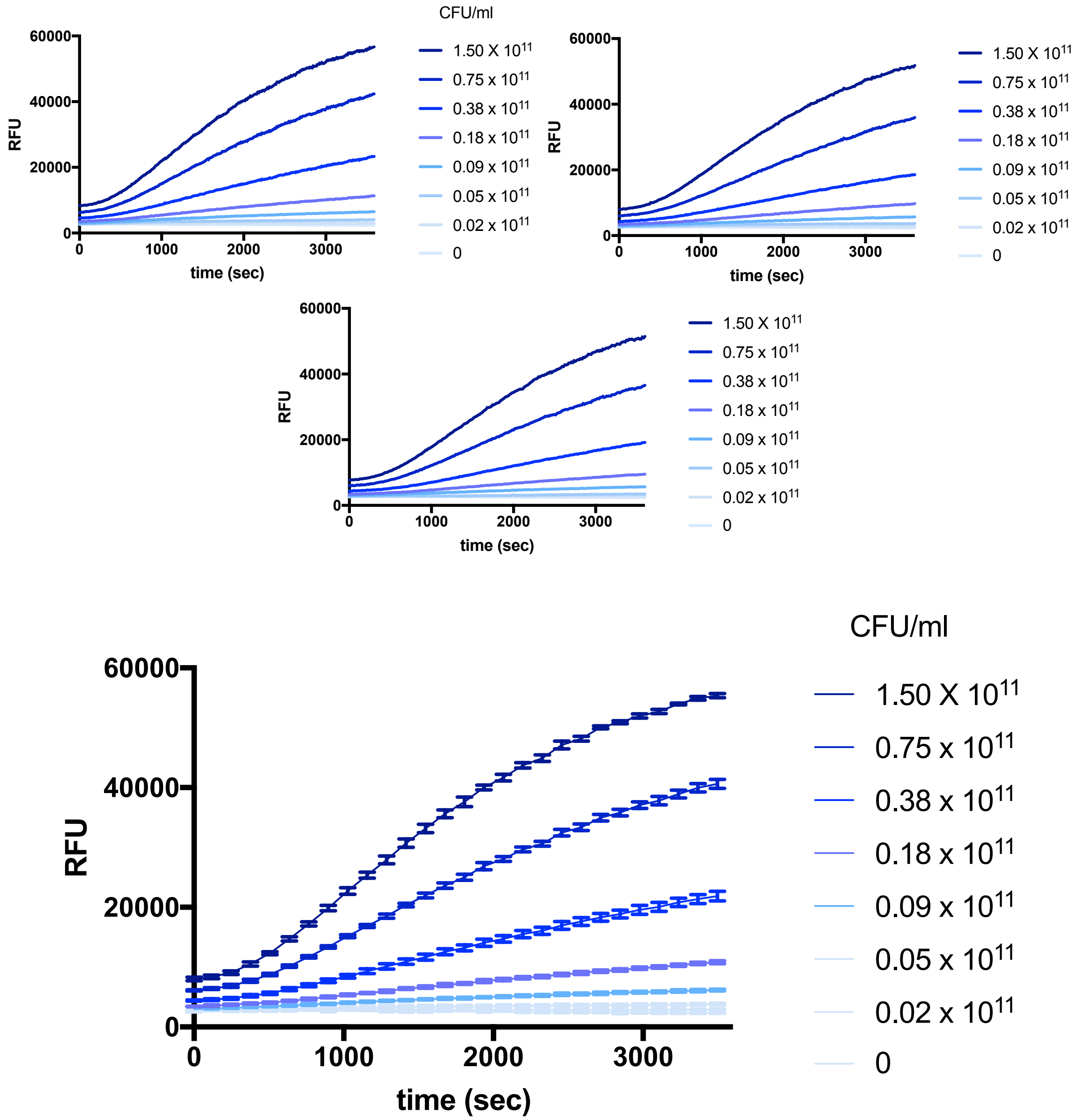

SI Figure 10. The CA-AMCA assay is capable of evaluating BSH activity *in vivo*. CA-AMCA (150 μM) was added to whole cell suspensions of *L. plantarum* of varying density, and reaction kinetics were followed through quantification of fluorescence (Ex: 350, Em: 450). A) Individual replicates. B) Averages for selected timepoints with standard deviations shown (n = 3 replicates).

A)

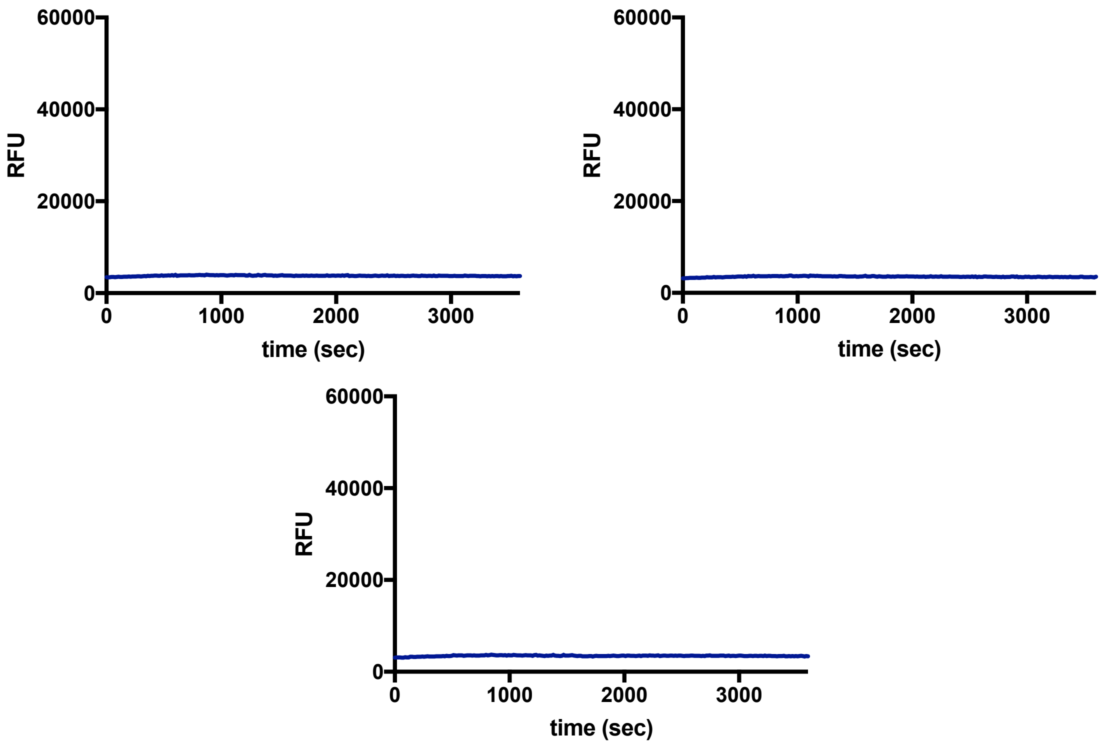

B)

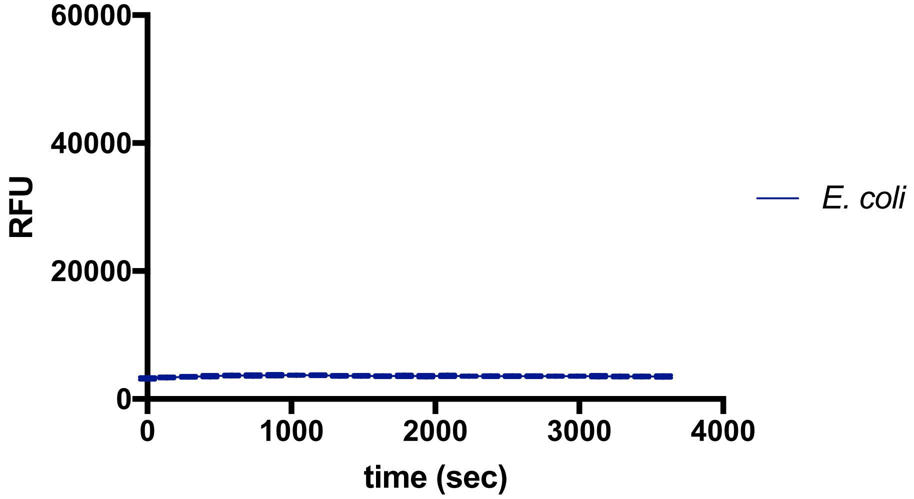

SI Figure 11. CA-AMCA is not turned over in cell lines that do not have BSH. Addition of CA-AMCA (150 μM) to whole cell suspensions of *E. coli*, which do not express BSH, does not cause an increase in fluorescence. The Y-axis range was set the same as the L. plantarum plots for ease of comparison. A) Individual replicates. B) Averages for selected timepoints with standard deviations shown (n = 3 replicates).

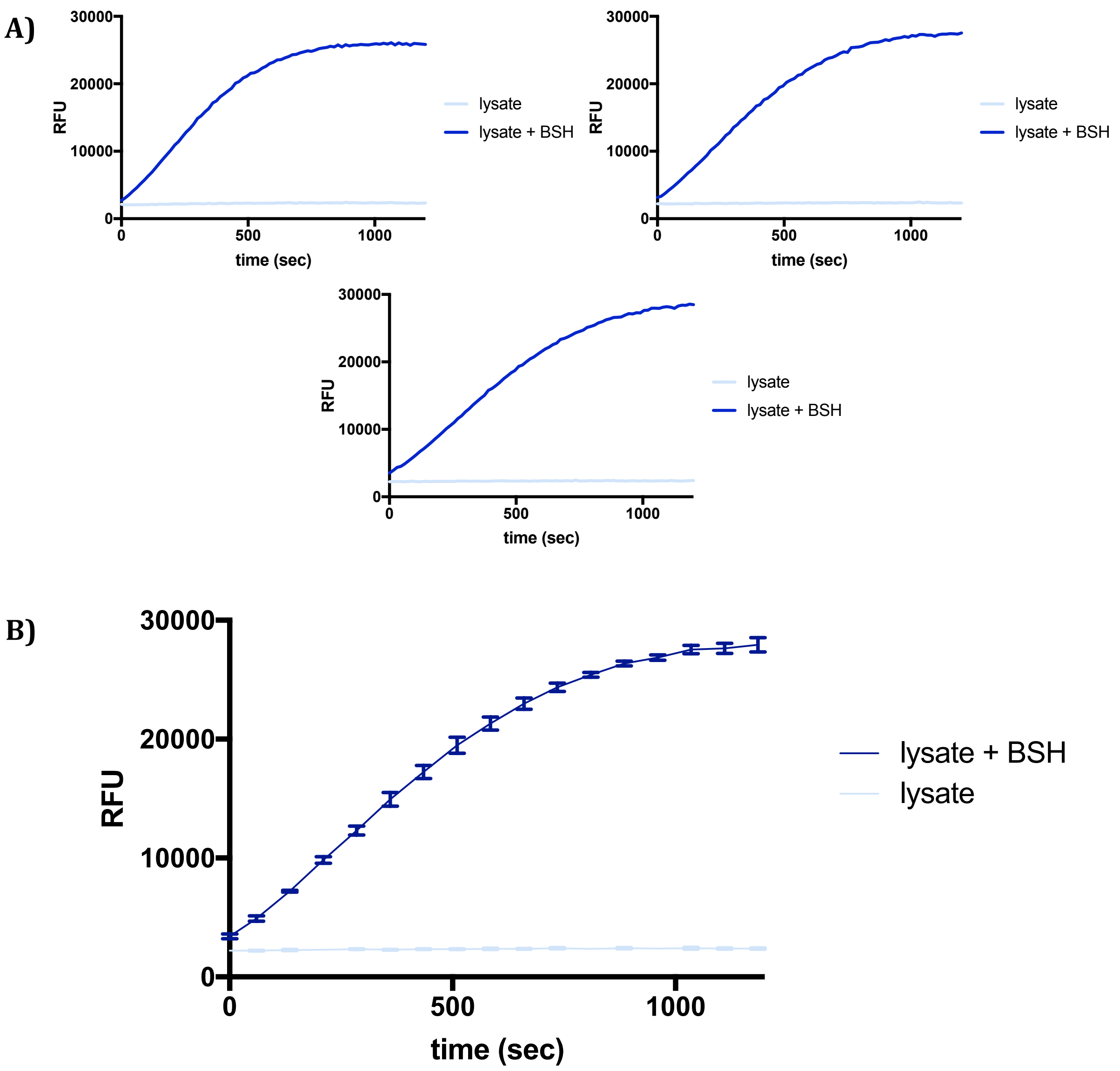

SI Figure 12. CA-AMCA assay reports on BSH activity in gut microbiome lysates. A) Individual replicates for purified BSH (20 μg/ml) added to gut microbiome lysate. B) Averages for selected timepoints with standard deviations shown (n = 3 replicates).

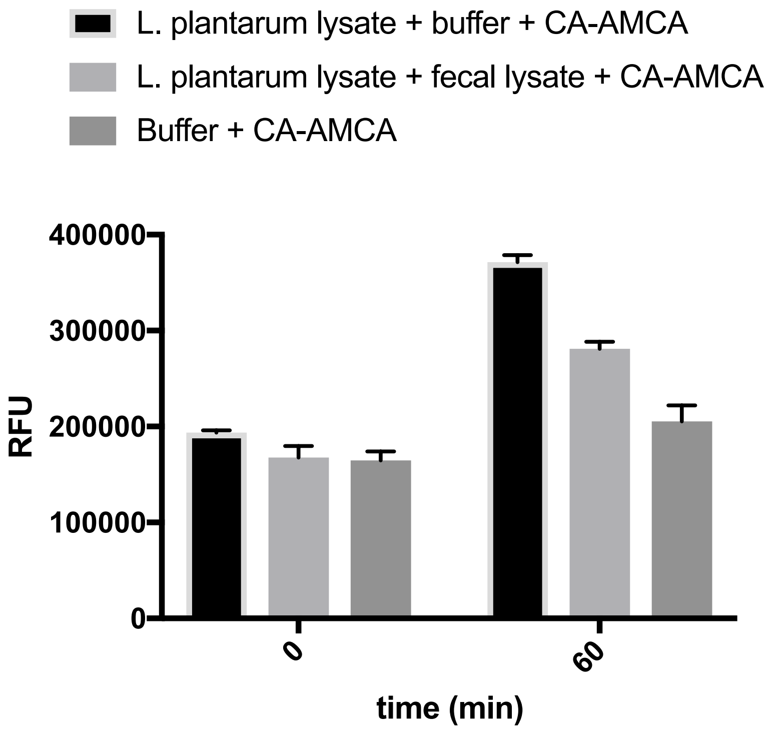

SI Figure 13. Signal for endogenously expressed BSH is lower when in the presence of fecal microbiome lysate. The signal from CA-AMCA (150 μM) when added to *L. plantarum* lysate (0.25 mg/ml) when added to fecal microbiome lysate (0.25 mg/ml), although attenuated relative to control. Longer incubation times were required to observe signal.

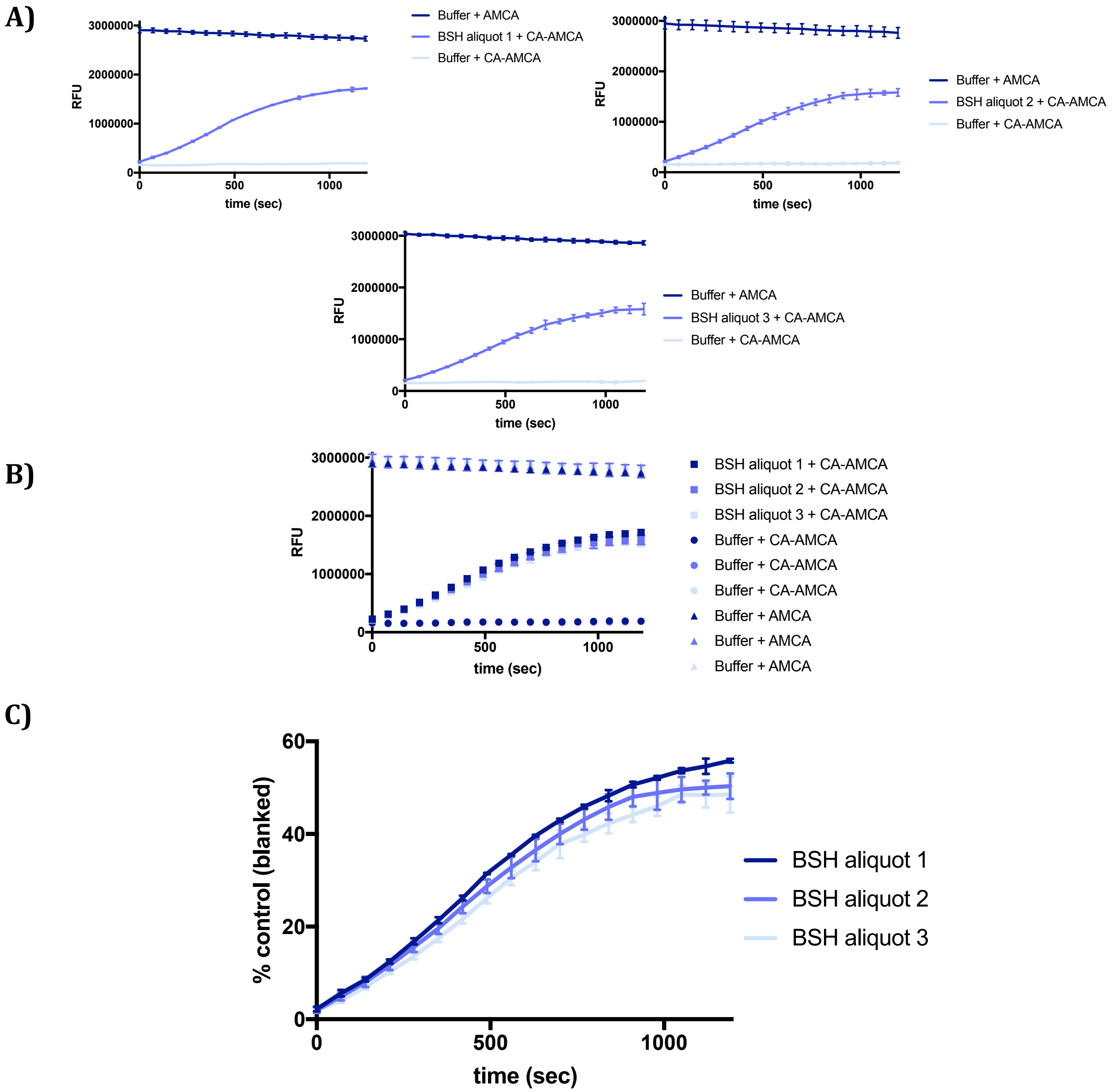

SI Figure 14. Characterization of different frozen BSH aliquots reveals low assay variability. Three separate aliquots of BSH from the same purification batch were analyzed to demonstrate assay variability. BSH (20 μg/ml) was co-incubated with CA-AMCA (150 uM) at 37°C for 20 minutes. 150 μM CA-AMCA in buffer alone, and 150 μM AMCA in buffer alone were both included as controls. Error bars (SD) for n = 3 replicates are shown over a connecting line, except for B, which is symbols for average values overlayed with error bars. A) Individual assay results for each aliquot. B) Overlay of assay results for each aliquot. C) Overlay of assay results for each aliquot that has been blanked with CA-AMCA in buffer alone, and normalized using 150μM AMCA in buffer alone.

A)

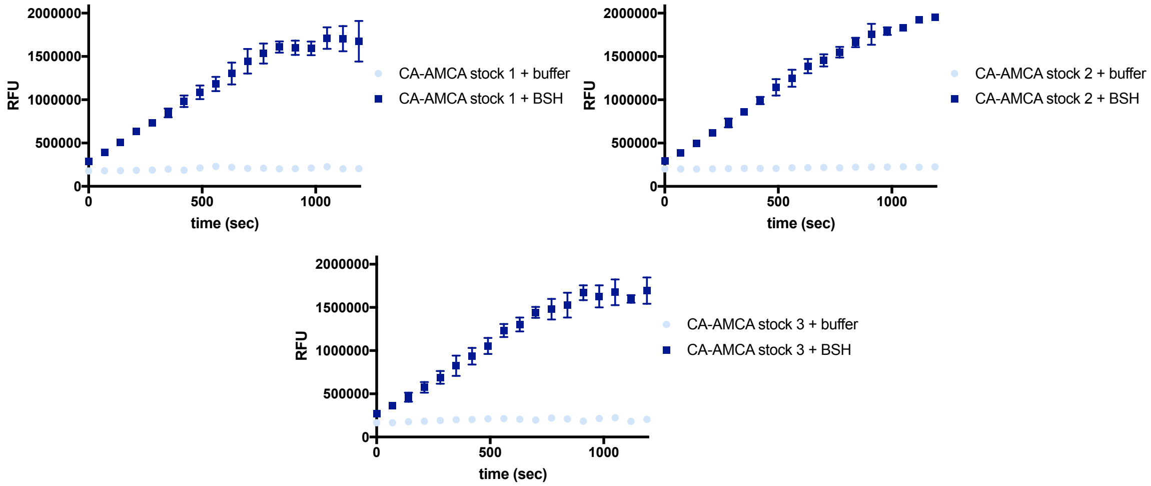

B)

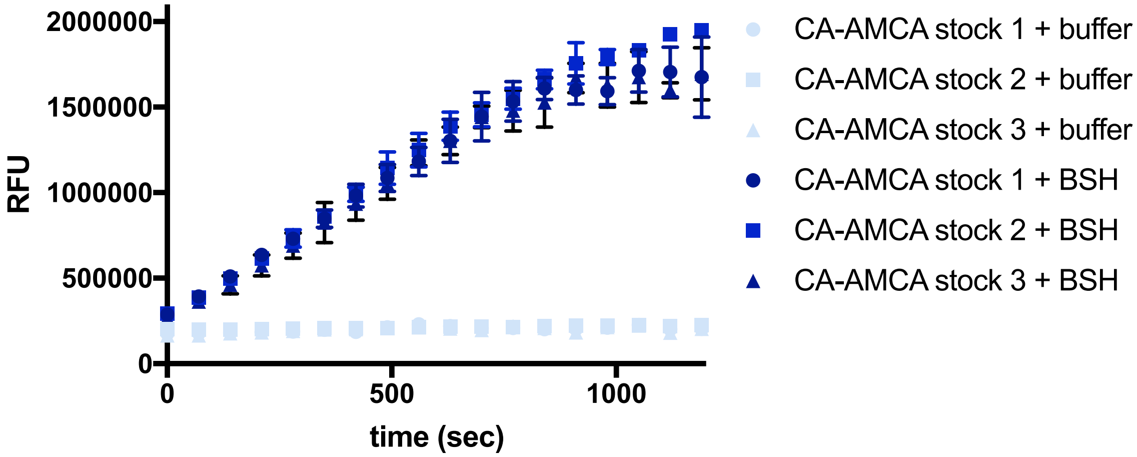

SI Figure 15. Low assay variability from separately prepared solutions of CA-AMCA tested with purified BSH. Three separate DMSO solutions of CA-AMCA were prepared using the purified solid. The three separately prepared CA-AMCA stock solutions were then tested against the same purified BSH sample. Each stock solution was tested with n = 3 replicates. Error bars for some points were smaller than the symbols, and cannot be seen. A) Experimental results from individual CA-AMCA preparations tested against BSH (n = 3). B) Overlay of all three runs.

II. SUPPLEMENTAL EXPERIMENTAL PROCEDURES

i. General synthetic methods

Unless otherwise noted, all reagents were obtained from commercial sources and used without further purification. All reagents and solvents were obtained from commercial suppliers and were used as is without further purification. Chemical shifts are reported in ppm (δ) referenced to the NMR solvent residual peak, and coupling constants (J) are in hertz. All reactions were monitored using TLC and LTQ-MS. For characterization of new compounds, 1H, 13C NMR and LTQ-MS data have been included. Mass Spectrometry (HRMS) was carried out at PNNL (Anil Shukla).

ii. Chemical synthesis

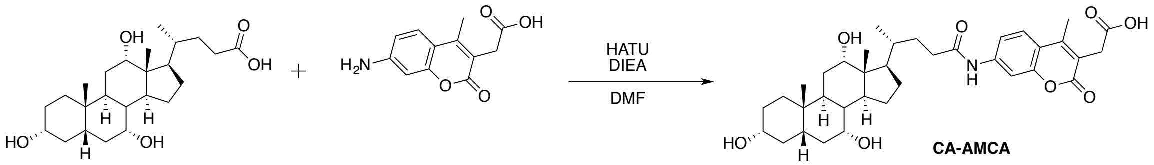

Synthesis of **CA-AMCA**. Cholic acid (120 mg, 0.295 mmol) and HATU (112.2 mg, 0.295 mmol), were added to an oven-dried round-bottomed flask charged with a stir bar, followed by DMF (2 ml) and diisopropylethylamine (310 µl). The reaction was allowed to stir for 15 minutes at room temperature, and then AMCA (137.7 mg, 0.59 mmol) was added. The reaction was heated to 50° C for 4 hrs. The reaction mixture was allowed to cool to room temperature, and then directly loaded onto a reverse phase HPLC column for purification (linear gradient of 10 → 95% CH_3CN in H_2O). The product was isolated as a pale orange solid (44.5 mg, 24.3% yield).

Spectral data. ^1H NMR (500 MHz, DMSO-*d6*): δ 10.30 (1H, s), 7.79-7.67 (2H, m), 7.51-7.43 (1H, m), 4.32-4.21 (1H, m), 4.11-4.04 (1H, m), 4.00-3.94 (1H, m), 3.81-3.73 (1H, m), 3.64-3.48 (3H, m), 3.21-3.10 (1H, m), 2.53-2.51 (1H, m), 2.42-2.08 (7H, m), 2.03-1.93 (1H, m), 1.87-1.70 (3H, m), 1.68-1.58 (2H, m), 1.47-1.10 (10H, m), 1.00-0.90 (4H, m), 0.80 (3H, s), 0.58 (3H, s); ^{13}C NMR (125 MHz, DMSO-*d6*) δ 176.18, 175.05, 173.32, 173.00, 171.92, 168.94, 161.35, 152.79, 149.02, 142.58, 126.47, 115.63, 115.53, 105.65, 71.47, 70.90, 66.70, 46.55, 41.99, 41.85, 40.92, 40.60, 35.78, 35.60, 35.37, 34.05, 33.44, 31.69, 30.87, 29.05, 27.73, 26.69, 23.27, 17.63, 15.36, 12.83. HRMS-ESI (*m/z*): $[M + Na]^+$ calcd for $C_{36}H_{49}NO_8$, 646.335; found 646.333.

iii. Spectral data

CA-AMCA:

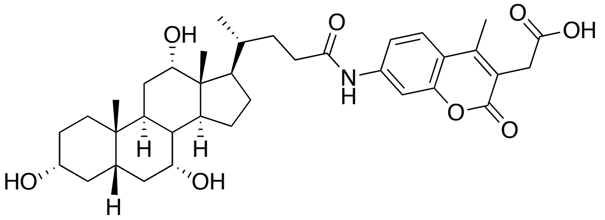

CA-AMCA ¹H:

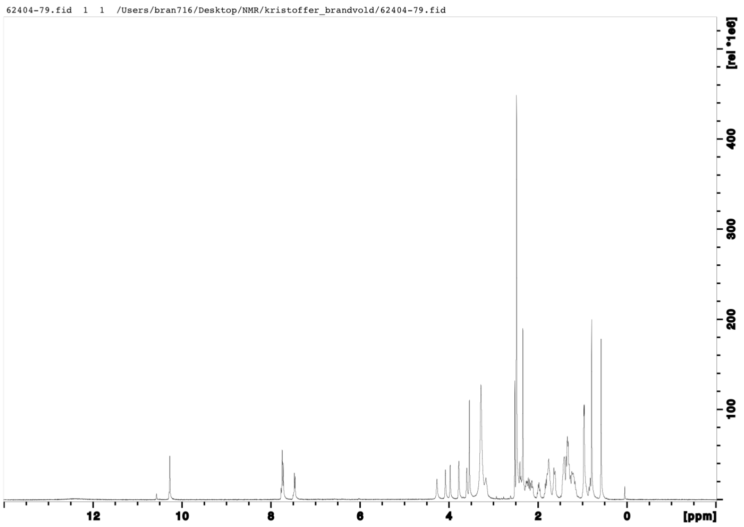

CA-AMCA ¹³C:

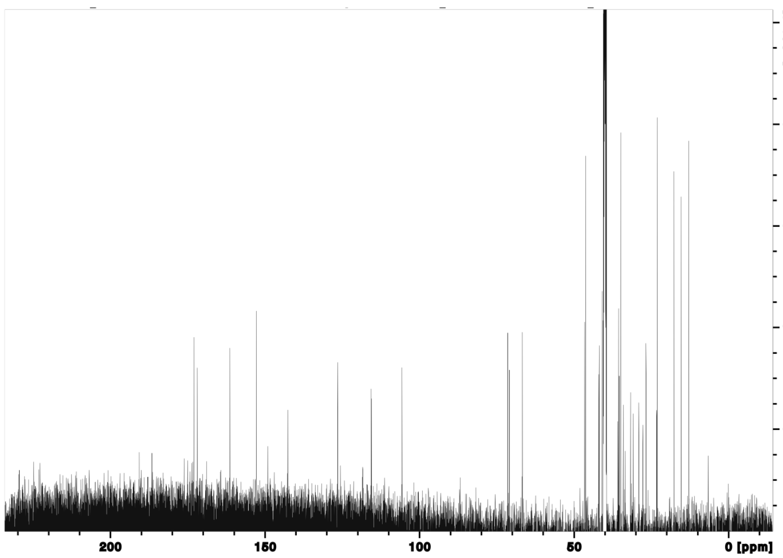

iv. Expression, purification and characterization of recombinant BSH

Production of *L. plantarum* Bsh1

The vector for Bsh1 production, pET32c-Bsh1 was constructed from pET32c (obtained from a laboratory stock) and *L. plantarum* WCFS1. Primers were ordered from Integrated DNA Technologies. pET32c-Bsh1 was constructed using Phusion Polymerase and Gibson Assembly (New England BioLabs) with the primers bsh1_F (5' – AACTTTAAGAAGGAGATATAATGTGTACTGCCATAACTTATC – 3'), bsh1_R (5' – TTGTTAGCAGCCGGATCTCATTAATGGTGATGGTGATGGTGGTTAACTGCATAGTATTGTGC – 3'), pET_F (5' – CACCATCACCATCACCATTAATGAGATCCGGCTGCTAAC – 3'), and pET_R (5' – TATATCTCCTTCTTAAAGTTAAACAAAATTATTTCTAGAGGGGAATTGTTATCCGCTC – 3'). Overnight cultures were diluted 1:200 into LB with ampicillin and grown at 37° C to OD_{600} = 0.6. Expression was induced overnight at 16° C via the addition of 1 mM IPTG. Protein was purified using the Ni-NTA agarose Purification System (ThermoFisher Scientific) per manufacturers protocol. After isolation, the buffer was exchanged into phosphate buffer using a 10 kDa MWCO filter (Amicon).

Protein purity

Purified bile salt hydrolase protein was prepared as described above and subjected to SDS PAGE in the specified concentrations. The gel was stained using GelCode Blue (Thermo Scientific).

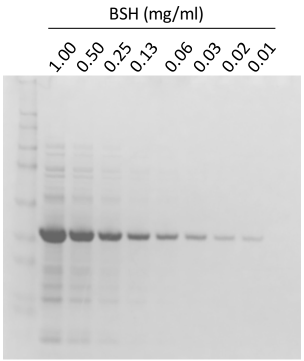

v. Biological characterization of probes

Purified BSH assay

Reaction volumes of 100 μL were used in Microfluor1 black flat bottom microtiter 96 well plates (Thermo Scientific). 50 μl BSH (80, 40, 20, 10 ,5, 2.5, 1.3, 0 μg/ml) in 0.1 M sodium phosphate buffer (pH = 6) were added to the appropriate wells. A 50 μL solution of probe (300 μM) in buffer with 5% DMSO was then added to initiate the reaction. Reactions were immediately placed in a plate reader (SPECTRAmax Gemini XS) pre-warmed to 37°C, and

reaction progress was monitored at 450 nm (excitation 350 nm) for 20 minutes. Reactions had final concentrations of 150 μM probe and 2.5% DMSO.

GCA and iodoacetamide competition assay
Experiments were run analogously to the purified BSH assay, except that competitors were added before CA-AMCA probe. Final concentrations were: BSH (10 μg/ml), DMSO (2.5% v/v), CA-AMCA (150 μM), and GCA (10mM) or IAA (12 mM), respectively.

***K*m determination**
Reaction volumes of 100 μL were used in Microfluor1 black flat bottom microtiter 96 well plates (Thermo Scientific). 50 μL of enzyme (40 μg/ml) in buffer (0.1 M sodium phosphate, pH = 6) was added to each well. 50 μL of appropriate CA-AMCA substrate dilution (2500, 1250, 625, 312.5, 156.2, 78.1, 39.1, 0 μM) dissolved in buffer with 5% DMSO was added to start the reaction. Reactions were immediately placed in a plate reader (SPECTRAmax Gemini XS) pre-warmed to 37°C, and reaction progress was monitored at 450 nm (excitation 350 nm) for 20 minutes. Reactions had final concentrations of 20 μg/ml enzyme, 0.1 M sodium phosphate (pH 6), and 2.5% DMSO.

The initial rate data was used for determination of K_m values. For K_m determination, the kinetic values were obtained directly from nonlinear regression of substrate-velocity curves in the presence of varying concentrations of BA-AMCA. The equation Y = (Vmax * X)/(Km + X*(1+X/Ki)), X = substrate concentration (μM) and Y = enzyme velocity (RFU/s); was used in the nonlinear regression. Each BA-AMCA K_m value was determined using at least three independent experiments.

***K*i determination**
Reaction volumes of 100 μL were used in Microfluor1 black flat bottom microtiter 96 well plates (Thermo Scientific). 50 μL of enzyme (10 μg/ml) in buffer (0.1 M sodium phosphate, pH = 6) was added to each well. A 50 μL solution of probe (50 μM) and CAPE (10000, 3333, 1111, 370, 123, 41, 14 μM) in buffer with 5% DMSO was then added to initiate the reaction. Reactions were immediately placed in a plate reader (SPECTRAmax Gemini XS) pre-warmed to 37°C, and reaction progress was monitored at 450 nm (excitation 350 nm) for 20 minutes. Reactions had final concentrations of 5 μg/ml enzyme, 0.1 M sodium phosphate (pH 6), and 2.5% DMSO.

HPLC assay
10 μL of 6 mM CA-AMCA in DMSO was mixed with 190 μL of .1 M sodium phosphate buffer (pH 6). 200 μL of enzyme in buffer was added to start the reaction. At varying time points (0, 1, 2, 3, 4, 5, 7.5, 10, 15, 20 minutes) 30 μL aliquots were removed and immediately mixed with 2 μL of 500 mM iodoacetamide to inactivate the hydrolase. After all samples were collected, 10 μL of each was loaded onto a C18 reverse-phase HPLC column (Phenomenex) on an Agilent 100 series analytical HPLC for characterization. A 5→100% MeOH in H_2O linear gradient over 15 minutes was used and elution stream was monitored at 450 nm (excitation 350 nm).

Purified BSH added to *E. coli* lysate

Reaction volumes of 100 µL were used in Microfluor1 black flat bottom microtiter 96 well plates (Thermo Scientific). 50 µl of *E. coli* lysate (2 mg/ml) with varying concentrations of BSH (80, 40, 20, 10 ,5, 2.5, 1.3, 0 µg/ml) in 0.1 M sodium phosphate buffer (pH = 6) were added to the appropriate wells. A 50 µL solution of probe (300 µM) in buffer with 5% DMSO was then added to initiate the reaction. Reactions were immediately placed in a plate reader (SPECTRAmax Gemini XS) pre-warmed to 37°C, and reaction progress was monitored at 450 nm (excitation 350 nm) for 20 minutes. Reactions had final concentrations of 150 µM probe and 2.5% DMSO. The *E. coli* strain used was DH5α.

***Lactobacillus plantarum* lysate assay**

Reaction volumes of 100 µL were used in Microfluor1 black flat bottom microtiter 96 well plates (Thermo Scientific). 50 µL of *L. plantarum* lysate (3000, 1500, 750, 375, 188, 94, 47 µg/ml) in buffer (0.1 M sodium phosphate, pH = 6) was added to each well. A 50 µL solution of probe (300 µM) in buffer with 5% DMSO was then added to initiate the reaction. Reactions were immediately placed in a plate reader (SPECTRAmax Gemini XS) pre-warmed to 37°C, and reaction progress was monitored at 450 nm (excitation 350 nm) for 20 minutes. Reactions had final concentrations of 150 µM probe, 0.1 M sodium phosphate (pH 6), and 2.5% DMSO. *L. plantarum* was purchased from ATCC; Strain ID NCIMB 8826 (WCFS1). ATCC catalog BAA-793.

Whole-cell *Lactobacillus plantarum* assay

A starter culture was created by inoculation of 5 ml MRS broth from a L. plantarum colony grown on MRS agar. The starter culture was incubated overnight at 37°C overnight. The 5 ml starter culture was then added to 95 ml fresh MRS broth and grown until the optical density at 600 nm reached 0.7. At that point, the cells were pelleted via centrifugation. The cell pellet was resuspended in 15 ml PBS, and centrifuged. The cells were additionally washed with PBS and pelleted once more. The resulting pellet was suspended in 380 µl PBS. The cell suspension was then serially diluted two-fold.

50 µl of the cell suspension was added to Microfluor1 black flat bottom microtiter 96 well plates (Thermo Scientific). A 50 µL solution of probe (300 µM) in buffer with 5% DMSO was then added to initiate the reaction. Reactions were immediately placed in a plate reader (SPECTRAmax Gemini XS) pre-warmed to 37°C, and reaction progress was monitored at 450 nm (excitation 350 nm) for 60 minutes. Reactions had final concentrations of 150 µM probe and 2.5% DMSO.

Preparation of human fecal microbiome cell lysate

Human fecal sample from a single donor was obtained from Lee BioSolutions (Cat no 991-18). Sample (1g) was suspended in 15 mL PBS with glass beads (3 mM) by vortexing for 30 seconds. The suspension was then allowed to rest on ice for 10 minutes to settle beads and large debris. The supernatant was transferred to a clean 50 mL conical tube. The supernatant was centrifuged at 700*g* for 15 minutes at 4°C. The supernatant was transferred to a clean 15 mL conical tube. The supernatant was then centrifuged at 7000*g* for 15 minutes at 4°C to

pellet the bacterial cells. The supernatant was then discarded. The cell pellet was resuspended in 1 mL cold PBS, and transferred to a 1.7 mL Eppendorf tube. The sample was vortexed briefly to homogenize the solution. The cells were then pelleted at 8000*g*. The pellet was then washed in 1 mL cold PBS. The pellet was then resuspended in 2 mL of PBS. Samples were transferred to 1.5 mL SafeLock tubes. 50-100 µL of small (0.3mm) glass beads were added. Samples were lysed in a Bullet Blender bead mill homogenizer (Next Advance; Model: BBX24B-CE) at max speed for 5 minutes. Bead beating was repeated two more times for a total of 3 rounds. Protein content was then determined by BCA assay (Thermo Scientific).

Fecal microbiome cell lysate assay with purified BSH added

Reaction volumes of 100 µL were used in Microfluor1 black flat bottom microtiter 96 well plates (Thermo Scientific). 50 µl of gut microbiome lysate (1 mg/ml) with BSH (40µg/ml) or buffer were added to the appropriate wells. A 50 µL solution of probe (300 µM) in buffer with 5% DMSO was then added to initiate the reaction. Reactions were immediately placed in a plate reader (SPECTRAmax Gemini XS) pre-warmed to 37°C, and reaction progress was monitored at 450 nm (excitation 350 nm) for 20 minutes. Reactions had final concentrations of 20 µg/ml BSH, 500 µg/ml lysate protein, 150 µM probe and 2.5% DMSO.

Fecal microbiome cell lysate assay with *L. plantarum* lysate added

Reaction volumes of 100 µL were used in Microfluor1 black flat bottom microtiter 96 well plates (Thermo Scientific). 25 µl of *L. plantarum* lysate (1 mg/ml) was added to 25 µl of fecal microbiome lysate (1 mg/ml) or buffer in the appropriate wells. A 50 µL solution of probe (300 µM) in buffer with 5% DMSO was then added to initiate the reaction. Reactions were immediately placed in a plate reader (Biotek Synergy H1) pre-warmed to 37°C, and reaction progress was monitored at 450 nm (excitation 350 nm) for 60 minutes. Reactions had final concentrations of 250 µg/ml *L. plantarum* lysate, 250 µg/ml fecal microbiome lysate, 150 µM probe and 2.5% DMSO.
